# Dexmedetomidine improved one-lung ventilation-induced cognitive dysfunction in rats

**DOI:** 10.1186/s12871-022-01658-w

**Published:** 2022-04-23

**Authors:** Mengyun Li, Zhe Jin, Jia Zhan, Yanlin Wang, Kai Chen

**Affiliations:** Department of Anesthesiology, Zhongnan Hospital of Wuhan University, Wuhan, Hubei 430071 PR China

**Keywords:** Dexmedetomidine, One-Lung Ventilation, CERO_2_, Cognitive Dysfunction, ERK1/2-CREB-Bcl-2

## Abstract

**Background:**

One-lung ventilation (OLV) is widely used in thoracic surgery. However, OLV may also increase CERO_2_ and aggravate delayed cognitive recovery. Here, we aimed to investigate the effect of dexmedetomidine (DEX) on cognitive function in rats undergoing OLV.

**Methods:**

Sprague-Dawley rats were randomly divided into two-lung ventilation (TLV) group, OLV group and OLV treated with DEX group. Group DEX received 25 μg/kg DEX i.p. 30 min before induction. After mechanical ventilation (MV), Morris water maze (MWM) test was carried out to examine spatial memory function. Western blotting was used to detect pERK1/2, pCREB, Bcl-2 and BAX in hippocampal tissues. Transmission electron microscopy (TEM) was used to observe the hippocampal CA1 region.

**Results:**

Post-MV, compared with group OLV, group DEX showed increases in percentage of target quadrant time (*P* < 0.05), platform crossings (*P* < 0.05), a reduction in CERO_2_ (*P* < 0.05), and pERK1/2, pCREB, and Bcl-2 significantly increased (*P* < 0.01 or *P* < 0.05), while BAX significantly decreased (*P* < 0.01), besides, a less damaged synaptic structure was observed in group DEX.

**Conclusions:**

DEX improved post-MV cognitive function in rats undergoing OLV, reduced cerebral oxygen consumption, protected synaptic structure and upregulated ERK1/2-CREB anti-apoptotic signaling pathway in hippocampal CA1 region.

**Supplementary Information:**

The online version contains supplementary material available at 10.1186/s12871-022-01658-w.

## Background

Cognitive impairments are common problems especially amongst older surgical patients [1]. These neurological complications termed as perioperative neurocognitive disorders (PND) [2], associate with poor functional recovery and increased mortality after major surgery [3]. The most commonly observed problems are memory impairment; difficulty in concentration and attention; and decreased information processing ability, such as visuospatial abstraction [4, 5]. Postoperative cognitive impairment can also lead to earlier retirement and greater utilization of social financial assistance [4]. Therefore, exploring the pathogenesis of PND and measures to improve early postoperative cognitive function is particularly important.

The mechanism of PND is complex, including intraneuronal neurofibrillary tangles and extracellular amyloid plaques, which cause central cholinergic system damage [6], neuroinflammation [7] and perioperative reduced cerebral oxygen saturation [8], among other issues. Nevertheless, current studies on the perioperative cerebral oxygen supply and demand balance are lacking. The central nervous system (CNS) and neurons in particular are highly susceptible to hypoxic-ischemic stress due to the lack of significant oxygen and energy reserves [9]. Even transient cerebral ischemia and hypoxia can increase the production of lactate [10], resulting in a change in the extracellular environment, which leads to damage to neurons and blood vessels. In the hypoxic environment, the electrophysiological activity of neurons will be irreversibly reduced, even leading to the death of neurons.

One-lung ventilation (OLV) is widely used in thoracic surgeries for better exposure of the surgical field. However, this nonphysiological ventilation mode inevitably leads to an imbalance in the ratio between ventilation and perfusion (V’_A_/Q’), aggravates the inflammatory reaction, and affects the oxygen supply of tissues. OLV may impair cerebral oxygen balance and induce POCD [11]. However, its specific biochemical mechanism, whether the balance of cerebral oxygen supply and demand changes during OLV, and whether there are effective interventions to prevent cognitive dysfunction caused by OLV remain to be further studied.

The common clinical indicators that reflect the balance of cerebral oxygen supply and demand are blood oxygen saturation of jugular vein bulb (SjvO_2_), arteriovenous oxygen difference (AVDO_2_) and cerebral oxygen extraction rate (CERO_2_) [12]. CERO_2_ refers to the percentage of oxygen extracted by neurons in the brain from arterial blood and accounts for changes in hemoglobin concentration. In general, CERO_2_ is approximately 30%. Increased CERO_2_ indicates increased cerebral oxygen uptake and insufficient cerebral blood flow relative to cerebral oxygen consumption. Decreased CERO_2_ indicates decreased cerebral oxygen uptake and excess cerebral blood flow relative to cerebral oxygen consumption.

Extracellular signal regulated kinase (ERK) is divided into ERK1 and ERK2, which are collectively known as ERK1/2, and their molecular weights are 44 kD and 42 kD, respectively. ERK is a mitogen-activated protein kinase (MAPK) [13]. The ERK signaling pathway is significantly associated with long-term potentiation (LTP) formation and learning and memory function [14]. Activated ERK entering the nucleus can promote phosphorylation of cAMP-response element binding protein (CREB) and other transcription factors to stimulate the expression of survivor-related genes and generate anti-apoptotic effects [15]. CREB is an important nuclear protein that selectively binds cAMP-response element (CRE) and regulates gene transcription. When CREB is phosphorylated to pCREB, its transcription ability will increase 10 to 20 times. CREB is involved in a variety of nervous system functions, such as regulation of synaptic plasticity, formation of long-term memory, survival and growth of neurons, and transduction of various intracellular signaling pathways [16]. pCREB can activate the transcription of relevant genes, such as Bcl-2, and mediate neuronal survival. This molecule is also closely related to synaptic formation and alleviating cognitive impairment after brain injury. ERK is a common pathway that promotes the phosphorylation of CREB and is involved in the consolidation of learning and memory in the hippocampus. ERK-mediated phosphorylation of CREB is involved in the formation of LTP and the maintenance of late LTP in hippocampal neurons [17].

The B cell lymphoma-2 (Bcl-2) gene family contains anti-apoptotic proteins represented by Bcl-2 and pro-apoptotic proteins represented by BAX. The hippocampus is recognized as a brain region closely related to spatial learning and memory. The CA1 area of the hippocampus is particularly sensitive to ischemia and hypoxia, and the learning and memory disorder caused by ischemia and hypoxia can be improved by inhibiting the apoptosis of hippocampal neurons. Activated ERK enters the nucleus, promotes CREB phosphorylation, induces Bcl-2 protein expression, and inhibits neuron apoptosis in the hippocampus [18].

Dexmedetomidine (DEX) is an α_2_-adrenoceptor agonist with sedative, anxiolytic, sympatholytic, and analgesic-sparing effects and minimal depression of respiratory function [19]. An increasing number of studies have proven that DEX can stabilize intraoperative hemodynamics, alleviate brain hypoxia-ischemia (HI) injury [20], and improve postoperative cognitive function [21]. In addition, DEX stimulates the α_2_ receptor and promotes the phosphorylation of extracellular signal-regulated kinase (ERK) [22], which plays an important role in cell plasticity and survival and may be related to the neuroprotective effect of DEX.

At present, there are few studies on the mechanism and intervention measures for cognitive dysfunction induced by OLV. To explore the effect of DEX on cognitive function after OLV and its possible biochemical mechanism, we constructed an OLV model in rats. The Morris water maze (MWM) [23] was used to evaluate the changes in post-OLV cognitive function with or without DEX from an ethological perspective. By monitoring the dynamic change in CERO_2_ during mechanical ventilation (MV), the effect of DEX on oxygen supply and demand balance in OLV rats was observed. Transmission electron microscopy (TEM) was used to observe the influence of DEX on the synaptic structure in the CA1 region of the hippocampus of OLV rats at the morphological level. Moreover, western blotting was used to detect the expression of pERK1/2, pCREB, Bcl-2 and BAX proteins in the hippocampus. The possible mechanism of DEX’s influence on post-OLV cognitive function was analyzed from the perspective of molecular biology, providing a theoretical basis for understanding the influence of OLV on postoperative cognitive function and the clinical application of DEX.

## Methods

### Animal

One hundred eight adult Sprague-Dawley (SD) rats (10–11 months old; 280 ± 20 g) were purchased from the medical experimental animal center of Hubei province and housed in standard conditions (room temperature, 22 °C; 12 h light/dark cycle) with free access to food and water and were acclimated to these conditions for at least 7 days prior to experiments. All experimental procedures were approved by the Animal Ethics Committee of Wuhan University, China, and were performed in accordance with the National Institutes of Health “Guidelines for the Care and Use of Laboratory Animals”. This study was approved by the animal ethics committee at the Zhongnan Hospital and Research Centre, Hubei, China and performed at animal experimental center of Wuhan University, China. The study was carried out in compliance with the ARRIVE guidelines. All methods are reported in accordance with ARRIVE guidelines (https://arriveguidelines.org) for the reporting of animal experiments.

### Experimental groups and treatments

**Fig. 1:**
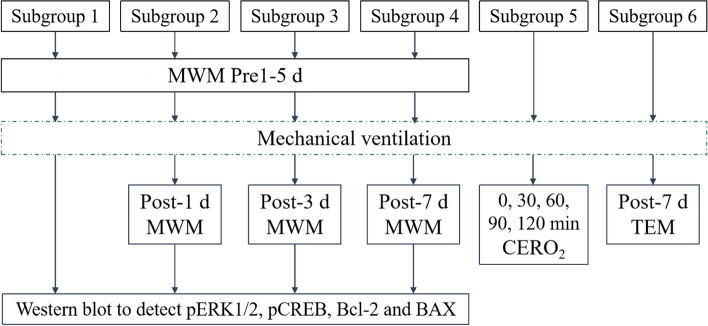
Experimental protocol. MWM, Morris water maze. CERO_2_, cerebral oxygen extraction rate. TEM, transmission electron microscopy

SD rats were randomly divided into 3 groups, and each group was divided into 6 subgroups (Fig. 1): TLV group (TLV group 1–6, *n* = 6), OLV group (OLV group 1–6, *n* = 6) and DEX group (DEX group 1–6, *n* = 6). In the TLV group, the same quantity of normal saline was administered by intraperitoneal injection 30 min before anesthesia, and TLV was administered for 120 min. In the OLV group, the same quantity of normal saline was administered by intraperitoneal injection 30 min before anesthesia, and OLV was administered for 90 min followed by TLV for 30 min. In the DEX group, 25 μg/kg DEX was administered by intraperitoneal injection 30 min before anesthesia, and OLV was administered for 90 min followed by TLV for 30 min. MWM training was conducted 5 days before MV (denoted as pre-5 days, pre-4 days, pre-3 days, pre-2 days and pre-1 day, respectively) in subgroups 1–4 at 14:00–16:00 every day and 1, 3 and 7 days after MV (denoted as post-1 day, post-3 days and post-7 days, respectively) in subgroups 2–4.

The right internal jugular vein and left common carotid artery were placed in the reverse flow direction before intubation after induction for the 5th subgroup in each main group. At 0, 30, 60, 90 and 120 min after MV (denoted as T_0_, T_1_, T_2_, T_3_ and T_4_, respectively), 0.2 ml blood from the right internal jugular vein and left common carotid artery was collected for blood gas analysis. CERO_2_ was calculated according to the Fick formula.

$${\mathrm{CERO}}_2=\left({\mathrm{CaO}}_2-{\mathrm{CjvO}}_2\right)/{\mathrm{CaO}}_2$$

$${\mathrm{CaO}}_2=\mathrm{Hba}\times \mathrm{l}.36\times {\mathrm{SaO}}_2+0.0031\times {\mathrm{PaO}}_2$$

$${\mathrm{CjvO}}_2=\mathrm{Hbjv}\times \mathrm{l}.36\times {\mathrm{SjvO}}_2+0.0031\times {\mathrm{PjvO}}_2$$

CaO_2_, Hba, SaO_2_ and PaO_2_ are the oxygen content, hemoglobin, oxygen saturation and oxygen partial pressure of carotid arterial blood, respectively.

CjvO_2_, Hbjv, SjvO_2_ and PjvO_2_ are the oxygen content, hemoglobin, oxygen saturation and oxygen partial pressure of carotid venous blood, respectively.

Seven days after MV, the CA1 region of the hippocampus of the 6th subgroup was taken for TEM to observe synaptic structure.

### Mechanical ventilation

OLV technology is widely used in thoracic surgery, which can provide good operating conditions for surgeons. However, the OLV process often leads to increased intrapulmonary shunt resulting in various pathophysiologic changes. Some studies have used deep endotracheal intubation to establish single-lung ventilation models in rats or rabbits.

Rats received general anesthesia with 1.5% isoflurane. Intratracheal intubation was performed under direct vision with a homemade endotracheal catheter comprising an animal urethral catheter with a balloon. The length was 15 cm, and the main lumen was used for ventilation. The side lumen was connected to the balloon. The depth of the catheter was 5 cm from the incisor to perform TLV. Rats were mechanically ventilated (TOPO; Kent Scientific, Torrington, CT 06790, USA) in pressure-controlled mode with V_T_ = 8 ml/kg, RR = 60 bpm, and fraction of inspired oxygen (FiO_2_) = 1.0.

**Fig. 2:**
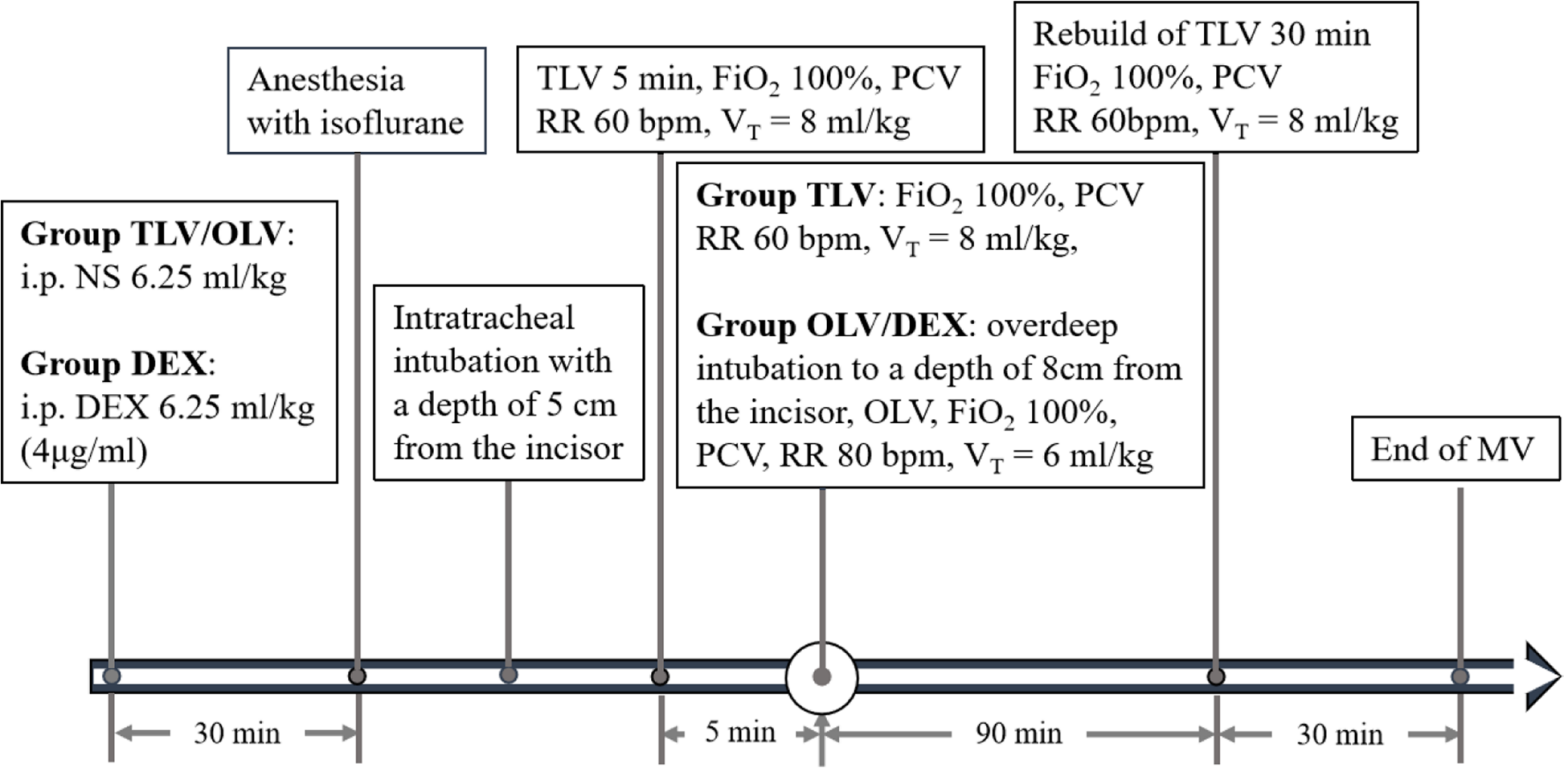
Realisation of mechanical ventilation. TLV, two-lung ventilation; OLV, one-lung ventilation; DEX, dexmedetomidine; FiO_2_, fraction of inspired oxygen; PCV, pressure-controlled ventilation; RR, respiratory rate; V_T_, tidal volume; MV, mechanical ventilation

This endotracheal catheter was inserted into the right main bronchus through overdeep insertion to establish OLV in the right lung. Before the study began, the depth of the catheter was measured at 8 cm from the incisor, and the side tube was inflated by 0.3 ml, which can effectively reach the OLV of the right lung. In addition, we further confirmed OLV in the right lung by observing chest fluctuation and applying an animal stethoscope. Rats were mechanically ventilated (TOPO; Kent Scientific, Torrington, CT 06790, USA) in pressure-controlled mode with V_T_ = 6 ml/kg, RR = 80 bpm, and FiO_2_ = 1.0 (Fig. 2).

### Morris water maze (MWM)

Hippocampus-dependent spatial memory function was assessed by the MWM. The MWM comprised a black circular pool (1.5 m in diameter and 50 cm in height) that contained 35 cm black dyed water (25 ± 1 °C) and was divided into four equal quadrants: NE, NW, SE and SW. A platform (15 cm in diameter) was hidden 1.5 cm below the water surface in the SW quadrant. Each rat was placed into the water from each of the four quadrants successively, and a maximum of 90 s was allowed to find the platform. If rats failed to find the hidden platform, they were guided to the platform and remained there for 10 s. Four consecutive training sessions were performed once a day for 4 days. On the fifth day, the platform was removed, and the rats were allowed to explore for 90 s. Finally, a video tracking program was used to output data for statistical analysis (SMART 3.0; Panlab, Spain).

### Western blotting analysis

After all rats were euthanized with decapitation, the hippocampus was quickly dissected and stored at − 80 °C until use. Samples were homogenized in lysis buffer and then centrifuged, and the supernatants were collected. Finally, the protein concentration was determined by a BCA protein assay kit (Aspen Biological; China). Protein samples were separated on 10% SDS-polyacrylamide gels and blotted onto nitrocellulose membranes. Following blocking with blocking buffer for 1 h, the membranes were incubated at 4 °C overnight with rabbit anti-phospho-ERK1/2 (1:1000; CST; USA), rabbit anti-phospho-CREB (1:1000; CST; USA), mouse anti-Bcl-2 (1:1000; TDY Biotech; China) and rabbit anti-BAX (1:1000; Bioss; China) antibodies. Then, the membranes were incubated with HRP goat anti-mouse or goat anti-rabbit or rabbit anti-goat secondary antibody (Aspen Biological; China) for 1 h. Finally, the protein bands were scanned using AlphaEaseFC (AlphaInnotech, USA). In this experiment, we used traditional methods in Western blot, namely protein separation by electrophoresis, electric transfer and immune detection, where marker is prestained protein marker. Blots were cut prior to hybridisation with antibodies.

### Transmission electron microscopy

The animals in the 6th subgroups were anesthetized with isoflurane. The thoracic cavities were opened and perfused intracardially with ice-cold saline, followed by perfusion with 4% paraformaldehyde fixative for 10 min. Coronal sections (150 μm thick) were cut on a vibratome, and the CA1 pyramidal layer and stratum radiatum were dissected. Briefly, microdissected areas were washed in 0.1 mmol/L sodium phosphate buffer and postfixed at room temperature for 1 h in 1% osmium tetroxide. Samples were then rinsed in ultrapure water, and tissue blocks were dehydrated at room temperature through graded ethanol from 30 to 100% for 10 min each, including 1% uranyl acetate in 70% ethanol for 40 min, and embedded in Epon epoxy medium (Momentive Specialty Chemicals/Hexion, Columbus, OH, USA). After 24 h, 120-nm sections were cut with an ultramicrotome (DuPont, Wilmington, DE, USA) and stained with 4% uranyl acetate for 20 min and 0.5% lead citrate for 5 min. Ultrastructural changes in synapses in the CA1 were observed under a Hitachi HT7700 TEM microscope (Hitachi, Tokyo, Japan). The identities of images were coded and revealed to the observer only after the data analysis was complete.

### Statistical analysis

Statistical analysis was performed with IBM SPSS software (version 21.0; IBM, Armonk, NY, USA). The results are expressed as the mean ± standard error of the mean. Statistical analysis was performed with analysis of variance followed by the Student-Newman-Keuls multiple-comparisons test for numerical data. Differences between two groups were assessed with Student’s *t*-test for normally distributed data and with the Mann-Whitney rank-sum test for nonnormally distributed data under the supervision of an expert statistician. Significance was set at *P* < 0.05.

## Results

### Morris water maze

**Fig. 3:**
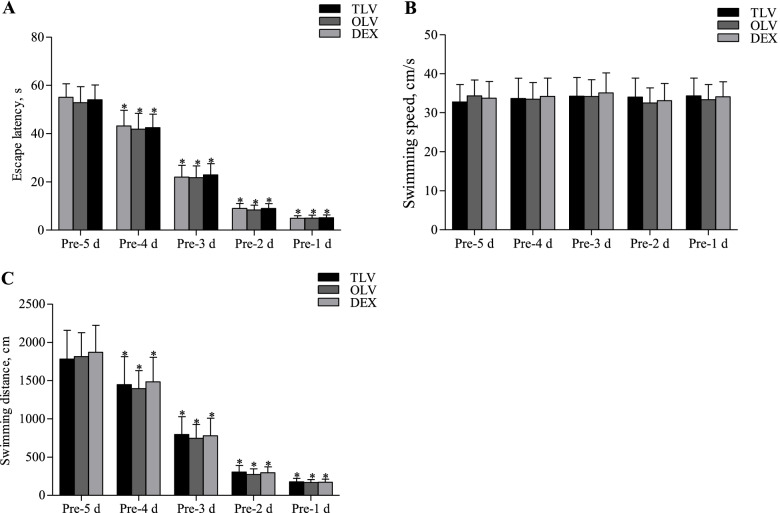
Comparison of pre-MV Morris water maze tests among three groups. *P < 0.01, as compared with pre-5 d

Pre-MV, rats were subjected to the MWM for 5 consecutive days. As the number of training days increased, the escape latency (EL) in each group was gradually reduced (*P* < 0.01, Fig. 3), the swimming speed was not significantly changed (Fig. 3), and the total swimming distance was reduced (*P* < 0.01, Fig. 3).

**Fig. 4:**
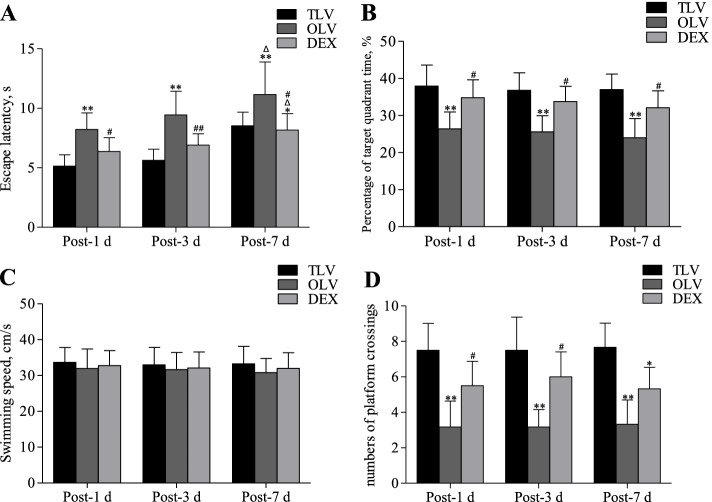
Comparison of post-MV Morris water maze tests among three groups. ^Δ^*P* < 0.05, as compared with post-1d; *P < 0.05, **P < 0.01, as compared with TLV group; ^#^*P* < 0.05, ^##^*P* < 0.01, as compared with OLV group

The escape latency was longer post-MV than pre-MV in each group. Compared with the TLV group, the OLV group showed significantly prolonged escape latency at 1, 3 and 7 days post-MV (Fig. 4, *P* < 0.01). The escape latency of the DEX group showed no significant difference at 1 day and 3 days post-MV and was prolonged at 7 days post-MV (Fig. 4, *P* < 0.05). Compared with the OLV group, the DEX group showed significantly shorter escape latency at each time point post-MV (Fig. 4, *P* < 0.01 or *P* < 0.05). There was no significant difference in escape latency post-MV within the groups. Post-MV swimming speed did not change significantly, and there was no significant difference between or within the groups (Fig. 4).

Compared with the TLV group, the OLV group showed a decrease in the percentage of target quadrant time at 1, 3 and 7 days post-MV (Fig. 4, *P* < 0.01). By contrast, the difference between the DEX and TLV groups was not significant. Compared with the OLV group, the DEX showed an increase in the percentage of target quadrant time post-MV (Fig. 4, *P* < 0.05). No significant difference was found in the percentage of target quadrant time within the groups’ post-MV.

Compared with the TLV group, the OLV group showed a significant reduction in platform crossings 1, 3 and 7 days after MV (Fig. 4, *P* < 0.05). Compared with the TLV group, the DEX group showed no significant difference in platform crossings 1 and 3 days post-MV but exhibited a significant decrease 7 days after MV (Fig. 4, *P* < 0.05). Compared with the OLV group, the DEX group showed a significant increase in platform crossings 1 and 3 days post-MV (Fig. 4, *P* < 0.05). Although the number of platform crossings increased in the DEX group 7 days after surgery, there was no significant difference. Moreover, there was no significant difference in the number of platform crossings 1, 3 and 7 days after MV within the groups.

### Cerebral oxygen extraction rate (CERO_2_)

**Fig. 5:**
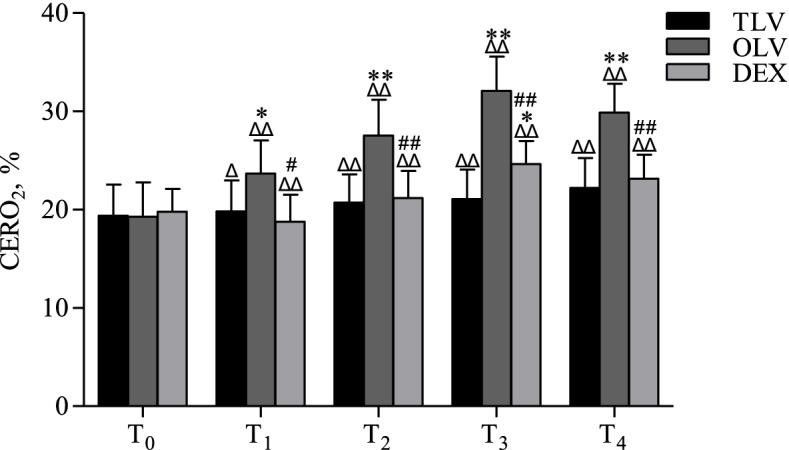
Comparison of CERO_2_ among three groups. T0, T1, T2, T3 and T4, 0, 30, 60, 90 and 120 min after MV, respectively. ^Δ^*P* < 0.05, ^ΔΔ^*P* < 0.01, as compared with T_0_; *P < 0.05, **P < 0.01, as compared with TLV group; ^#^*P* < 0.05, ^##^*P* < 0.01, as compared with OLV group

With the extension of MV time, CERO_2_ gradually increased in the TLV group. In the OLV and DEX groups, CERO_2_ increased with the duration of OLV and peaked at T_3_ (Fig. 5, *P* < 0.01). After the restoration of TLV, CERO_2_ decreased.

At T_0_, CERO_2_ showed no significant difference between the groups. At T_2_ and T_3_, as the duration of OLV increased, CERO_2_ in the OLV group was significantly increased compared with that in the TLV group (Fig. 5, *P* < 0.01). After TLV was restored in the OLV group, CERO_2_ decreased, but it remained higher than that in the TLV group at the same time point. In the DEX group, at the early stage of OLV, CERO_2_ was similar to that in the TLV group but was higher than that in the TLV group at the end of OLV (Fig. 5, *P* < 0.05). Moreover, CERO_2_ in the DEX group was significantly lower than that in the OLV group during the period of OLV and after the restoration of TLV.

### Western blotting

**Fig. 6:**
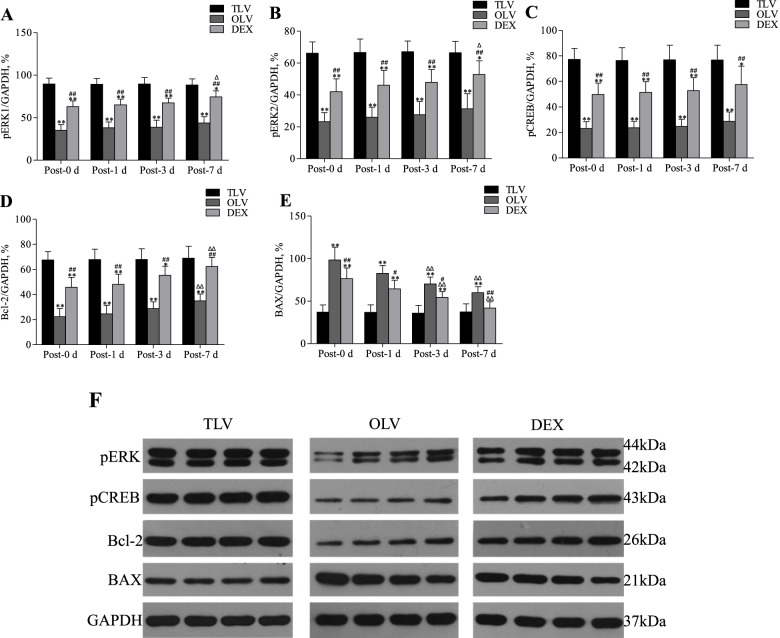
Comparison of pERK1, pERK2, pCREB, Bcl-2 and BAX in Hippocampal CA1 region post-MV among three groups. ^Δ^*P* < 0.05, ^ΔΔ^*P* < 0.01, as compared with post-0d in same group; *P < 0.05, **P < 0.01, as compared with TLV group at the same time point; ^#^*P* < 0.05, ^##^*P* < 0.01, as compared with OLV group at the same time point

In the TLV group, there was no significant change in the expression of pERK1, pERK2, pCREB and Bcl-2 proteins post-MV (Fig. 6). In the OLV group, the expression of pERK1, pERK2, pCREB and pCREB proteins post-MV gradually increased; nevertheless, only the difference in pCREB between the post-7 day and post-0 day subgroups was significant. The expression of BAX post-MV decreased gradually; compared with BAX post-0 days, BAX post-3 days and BAX post-7 days were significantly decreased. In the DEX group, the expression of pERK1, pERK2, pCREB and Bcl-2 proteins post-MV gradually increased, and the difference in pERK1, pERK2 and Bcl-2 between the post-7 day group and post-0 day group was significant. The expression of BAX post-MV gradually decreased, and compared with BAX post-0 days, BAX post-3 days and BAX post-7 days were significantly decreased.

Compared with the TLV group, the OLV group showed significant decreases in pERK1, pERK2, and pCREB and significant increases in BAX at all time points after MV. Compared with the TLV group, the DEX group showed significant decreases in pERK1, pERK2, and pCREB proteins at all time points after MV; at post-0 days, post-1 days and post-3 days, the level of Bcl-2 proteins in the DEX group significantly decreased, and BAX significantly increased. Compared with the OLV group, the DEX group exhibited significantly higher expression of pERK1, pERK2, pCREB and Bcl-2 proteins and significantly lower expression of BAX protein at all time points after MV.

### Transmission electron microscopy (TEM)

**Fig. 7:**
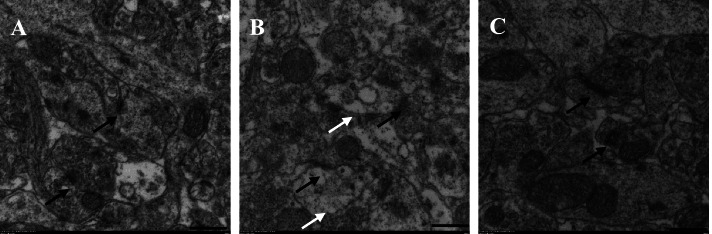
Chemical synapses in the CA1 region of rat hippocampus in three groups (10000×). Black arrows indicate chemical synapses, and white arrows indicate changes described. A, B and C were respectively TLV group, OLV group and DEX group. In A, the neurons were intact and the synaptic structure was complete. In B, a large number of vacuoles appeared in neurons, and part of the capsule was broken. In C, the neurons are intact and the synaptic structure is complete

**Fig. 8:**
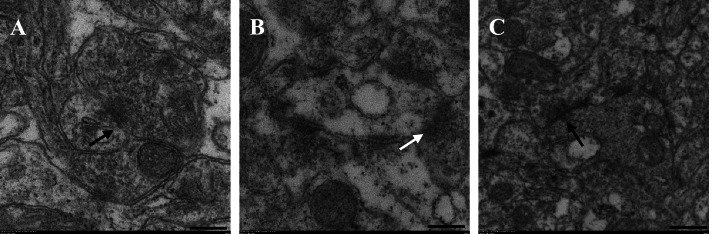
Chemical synapses in the CA1 region of rat hippocampus in three groups (20000×). Black arrows indicate chemical synapses, and white arrows indicate changes described. A, B and C were respectively TLV group, OLV group and DEX group. The complete synaptic structure can be seen in A, and the presynaptic membrane contains A large number of synaptic vesicles. In B, some synapses were broken, with few synaptic vesicles and fuzzy synaptic connections. The complete synaptic structure was observed in C, and the presynaptic membrane contained synaptic vesicles

Animals were killed immediately after MV, and the synaptic structure of the hippocampal CA1 region was observed by TEM (Figs. 7 and 8). In the TLV and DEX groups, neurons and neuroglia were intact, and the synaptic structure was more complete than that in the OLV group. However, fewer synaptic vesicles were observed in the DEX group than in the TLV group. Compared with the TLV group, the OLV group showed an increase in PSD thickness, and a decrease in the number of synaptic vesicles. Moreover, in the OLV group, vacuoles appeared, some neurons were swollen and ruptured, and the synaptic structure was incomplete.

## Discussion

To gain insight into the mechanisms underlying the negative effect of OLV on cognitive function and the possible therapeutic effect of DEX, we compared behavioral outcomes and parameters for the balance between cerebral oxygen supply and consumption, intraneuronal pathways, and neurosynaptic structure after TLV, OLV and OLV with DEX pretreatment. We hypothesized that OLV would be associated with more severe cognitive dysfunction, alterations in intraneuronal pathways and synaptic destruction. In previous clinical studies, our research group has also observed a higher incidence of delayed neurocognitive recovery among patients with OLV. However, there are limitations and many surgical interference factors in clinical studies; thus, it is difficult to compare OLV with TLV and determine the specific molecular biological mechanism. To avoid other risk factors affecting neurocognitive recovery, such as aging and surgical interference, we selected adult male SD rats aged 11–12 months that did not undergo surgery.

### Cognition and behavior after OLV

In concurrence with previous studies, we observed a decline in cognitive function in rats that underwent OLV [11, 24] and an improvement in cognitive function in rats that were treated with DEX [21]. We came to these conclusions as rats in the OLV group showed impaired performance and rats in the DEX group showed improved performance in the MWM.

After 5 days of training pre-MV, rats gradually formed a spatial memory of the environmental markers and the platform, which was consolidated and strengthened as the number of trainings increased. The MWM results showed that the post-MV escape latency was longer than the pre-MV escape latency in each group. Compared with the TLV group, the OLV group showed significantly longer escape latency, shorter target quadrant time and fewer platform crossings 1, 3 and 7 days post-MV. These results indicated decreased spatial memory of the same environmental markers and platform and the development of cognitive dysfunction in the OLV group. At 1, 3 and 7 days post-MV, rats in the DEX group had shorter escape latency, longer target quadrant residence time, and more platform crossings than did rats in the OLV group. Based on these findings, DEX protected postoperative spatial memory and could improve cognitive function post-OLV. Compared with the TLV group, the DEX group showed no difference in the post-MV escape latency 1 and 3 days post-MV. However, 7 days post-MV, the escape latency was longer in the DEX group than in the TLV group, indicating that DEX could ameliorate post-OLV cognitive function to some extent but could not completely reverse the cognitive decline caused by OLV.

### Cerebral oxygen consumption during mechanical ventilation

OLV, a nonphysiological ventilation mode, causes some blood to enter the left heart without oxygenation, further leading to the imbalance of V’A/Q’. Tang et al. [24] studied changes in cerebral oxygen saturation and influencing factors during OLV in patients undergoing thoracic surgery. The authors observed a significant decreased cerebral oxygen saturation during OLV. Insufficient cerebral oxygen supply may lead to neuronal apoptosis. Moreover, decreased perioperative cerebral oxygen saturation is associated with the pathogenesis of postoperative cognitive impairment [25].

CERO_2_ is a good indicator to reflect the balance between the supply and demand of cerebral oxygen because it accounts for the influence of hemoglobin concentration. Therefore, in this study, the effect of OLV on cerebral oxygen supply and demand balance was observed by detecting CERO_2_.

In this study, at the beginning of ventilation, CERO_2_ in the OLV group was the same as that in the TLV group. With the extension of ventilation time, CERO_2_ increased in each group, indicating that the oxygen supply of brain tissue decreased during MV. Compared with the TLV group, the OLV group showed a faster rise in CERO_2_. At T_2_ and T_3,_ CERO_2_ was significantly higher in the OLV group than in the TLV group, suggesting that the oxygen supply of brain tissue was relatively more insufficient during OLV. After TLV was restored, CERO_2_ decreased, and oxygen supply was improved.

Detection of CERO_2_ showed that DEX could significantly reduce CERO_2_ during OLV. CERO_2_ in the DEX group was only higher than that in the TLV group at the end of OLV. This result indicated that DEX can, to some extent, decrease the oxygen consumption of brain tissues during OLV and alleviate the insufficient oxygen supply, even though it could not reach the level of TLV.

### Neuronal apoptosis induced by OLV

ERK1/2-CREB-Bcl-2 is a classical anti-apoptotic signaling pathway [26] involving multiple proteins associated with cognitive function. Activation of the ERK pathway is involved in the formation of short-term memory and the transformation of short-term memory into long-term memory, namely, the process of memory consolidation [14]. Hypoxia leads to downregulation of ERK expression levels [27] and affects the synaptic plasticity regulated by ERK [28, 29].

CREB is mainly located in the nucleus, and its pro-transcriptional activity increases 10–20 times after activation to pCREB. CREB is involved in a variety of nervous system functions, such as regulation synaptic plasticity during development and adulthood [30], the survival and growth of neurons, and the transduction of various intracellular signaling pathways [31]. Additionally, CREB directly participates in the formation of spatial learning and memory in the MWM [32]. The activation of MAPK/ERK in hippocampal circuitry is required for consolidation and reconsolidation of recognition memory, and the activation of CREB by phosphorylation is crucial for the maintenance of LTP, which is crucial for memory formation [17, 26].

The Bcl-2 family of proteins consists of both prosurvival (e.g., Bcl-2, Bcl-XL, and Mcl-1) and prodeath (e.g., BAX, Bad, and PUMA) members [33]. Bcl-2 plays an important role in the inhibition of apoptosis. The currently known mechanisms include inhibiting intracellular calcium overload [34, 35], blocking caspase-dependent intrinsic apoptosis [36], blocking the apoptosis inducing factor (AIF)-dependent neuronal apoptosis pathway [37], and inhibiting ROS production by antioxidant stress. The hippocampus is critical to spatial learning and memory formation and is sensitive to hypoxia and ischemia. By inhibiting the apoptosis of hippocampal neurons, spatial learning and memory formation disorders caused by hypoxia and ischemia can be improved [38]. Activated ERK entering the nucleus can promote CREB phosphorylation, induce Bcl-2 protein expression, inhibit the apoptosis of neurons in the hippocampus and affect early postoperative cognitive function [36].

In various studies, DEX reduced the incidence of postoperative cognitive decline in aged patients via inhibition of inflammatory responses and protected traumatically injured hippocampal cells via a decrease in neuronal apoptosis [39]. Decreased learning and memory ability induced by splenectomy in adult mice was improved by DEX treatment (15 μg/kg or 25 μg/kg) prior to splenectomy, with significant improvement induced by 25 μg/kg DEX on day 3 (*P* < 0.05) [21]. Therefore, in this study, 25 μg/kg DEX was administered by intraperitoneal injection 30 min prior to induction.

In this study, western blotting was used to detect the expression levels of related proteins in the ERK1/2-CREB-Bcl-2 anti-apoptosis signaling pathway immediately after MV (T_1_) and T_2_, T_3_, and T_4_ post-MV.

In the OLV group, at T_1_, T_2_, T_3_ and T_4,_ the expression levels of pERK1/2, pCREB and Bcl-2 proteins in the hippocampus were lower than those in the TLV group, suggesting that the relatively insufficient oxygen supply to brain tissue caused by OLV inhibited the expression of related proteins in the anti-apoptotic ERK1/2-CREB-Bcl-2 signaling pathway. Detection at T_1_, T_2_, T_3_ and T_4_ in the OLV group showed that the expression levels of pERK1/2, pCREB and Bcl-2 proteins gradually increased over time post-MV, but only the expression levels of Bcl-2 protein showed significant differences, which may be affected by the sample size. Nevertheless, over time, the anti-apoptotic pathway is gradually restored. By comparing the expression of BAX protein in the 2 groups, we found that the pro-apoptosis pathway was activated after OLV, but the pro-apoptosis process gradually weakened over time.

In OLV, the expression levels of pERK1/2, pCREB and Bcl-2 were upregulated at the end of ventilation and 1, 3 and 7 days after ventilation, indicating that pretreatment with DEX activated the anti-apoptosis ERK1/2-CREB-Bcl-2 signaling pathway. BAX expression was downregulated at the end of ventilation and 1, 3 and 7 days after ventilation, indicating that pretreatment with DEX inhibited the pro-apoptosis pathway. This finding was consistent with previous studies showing that DEX can promote the phosphorylation of ERK and regulate the balance between apoptotic and anti-apoptotic proteins. Compared with the TLV group, as a result of OLV, the DEX group showed lower levels of upstream pERK1/2 and pCREB at 0, 1, 3 and 7 days after ventilation. By contrast, Bcl-2 reached the level of the TLV group 7 days after ventilation, suggesting that DEX had a limited anti-apoptotic effect. In the OLV group, the inhibition of the ERK1/2-CREB-Bcl-2 anti-apoptotic signaling pathway gradually weakened over time post-ventilation.

The CA1 region of the hippocampus is involved in the formation of learning and spatial memory, and its synaptic plasticity is related to the formation and maintenance of LTM. In this study, the effect of OLV on synapses in the hippocampal CA1 region was observed by TEM. Under TEM, the complete synaptic structure, including the presynaptic membrane, postsynaptic membrane and synaptic cleft, can be seen in the normal hippocampal CA1 region. Synaptic vesicles, mitochondria and other organelles can be seen in the presynaptic membrane, and a large number of electron-dense materials are attached to the surface of the pre- and post-synaptic membranes and the posterior membrane, showing a banded distribution. In the TLV group, the synaptic structure was intact in the hippocampal CA1 region, with visible pre- and post-synaptic membranes, concentrated electron-dense materials, and many visible intact synaptic vesicles in the presynaptic membrane. By contrast, in the OLV group, the neuronal density was reduced, vacuoles appeared in the cytoplasm, some neurons were necrotic, swelling and rupture occurred, synaptic structure was incomplete, synaptic membranes were partially broken, the electron-dense area was slightly blurred, and synaptic vesicles were reduced or absent. This result indicated that OLV caused a decrease in synaptic plasticity in the hippocampal CA1 region in rats as well as neuronal destruction and apoptosis. In the DEX group, the membranes of neurons and neuroglia in the hippocampal CA1 area were intact, the synaptic structure was more complete than that in the OLV group, the electron dense area was concentrated, and the number of synaptic vesicles was less than that in the TLV group but significantly better than that in the OLV group. Therefore, DEX can play a neuroprotective role in OLV rats.

## Conclusions

Our present study found that OLV can result in delayed neurocognitive recovery, and the main cause was insufficient cerebral oxygen supply during OLV. OLV inhibited the expression of anti-apoptotic proteins involved in the ERK1/2-CREB-Bcl-2 signaling pathway, induced the expression of pro-apoptotic protein BAX (which affected the synaptic plasticity of the hippocampal CA1 region), and even led to the apoptosis of hippocampal neurons. DEX improved early postoperative cognitive decline in OLV rats to some extent, and its mechanism included improving oxygen supply to brain tissue during OLV, stabilizing synapses in the hippocampal CA1 region, and upregulating the anti-apoptotic ERK1/2-CREB-Bcl-2 signaling pathway.

## Supplementary Information

**Additional file 1.**

## Data Availability

The datasets used and analysed during the current study are available from the corresponding author on reasonable request.

## Abbreviations

OLV: One-lung ventilation
DEX: Dexmedetomidine
PND: Perioperative neurocognitive disorders
CERO_2_: Cerebral oxygen extraction rate
ERK: Extracellular signal regulated kinase
Bcl-2: B cell lymphoma-2
