# Supplementary figures and images for: Dexmedetomidine improved one-lung ventilation-induced cognitive dysfunction in rats
Source: BMC Anesthesiol. 2022 Apr 23;22:115. doi: 10.1186/s12871-022-01658-w (PMC9034634; doi:10.1186/s12871-022-01658-w)

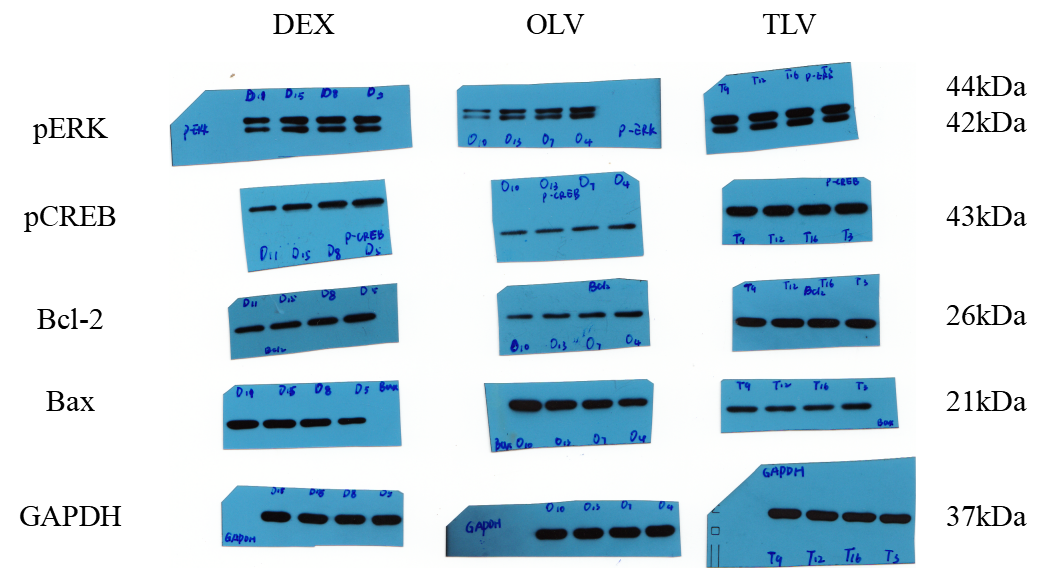

Supplement: Supplementary file 1 — Additional file 1. [file 12871_2022_1658_MOESM1_ESM.zip › original data with explanation 1.tif]

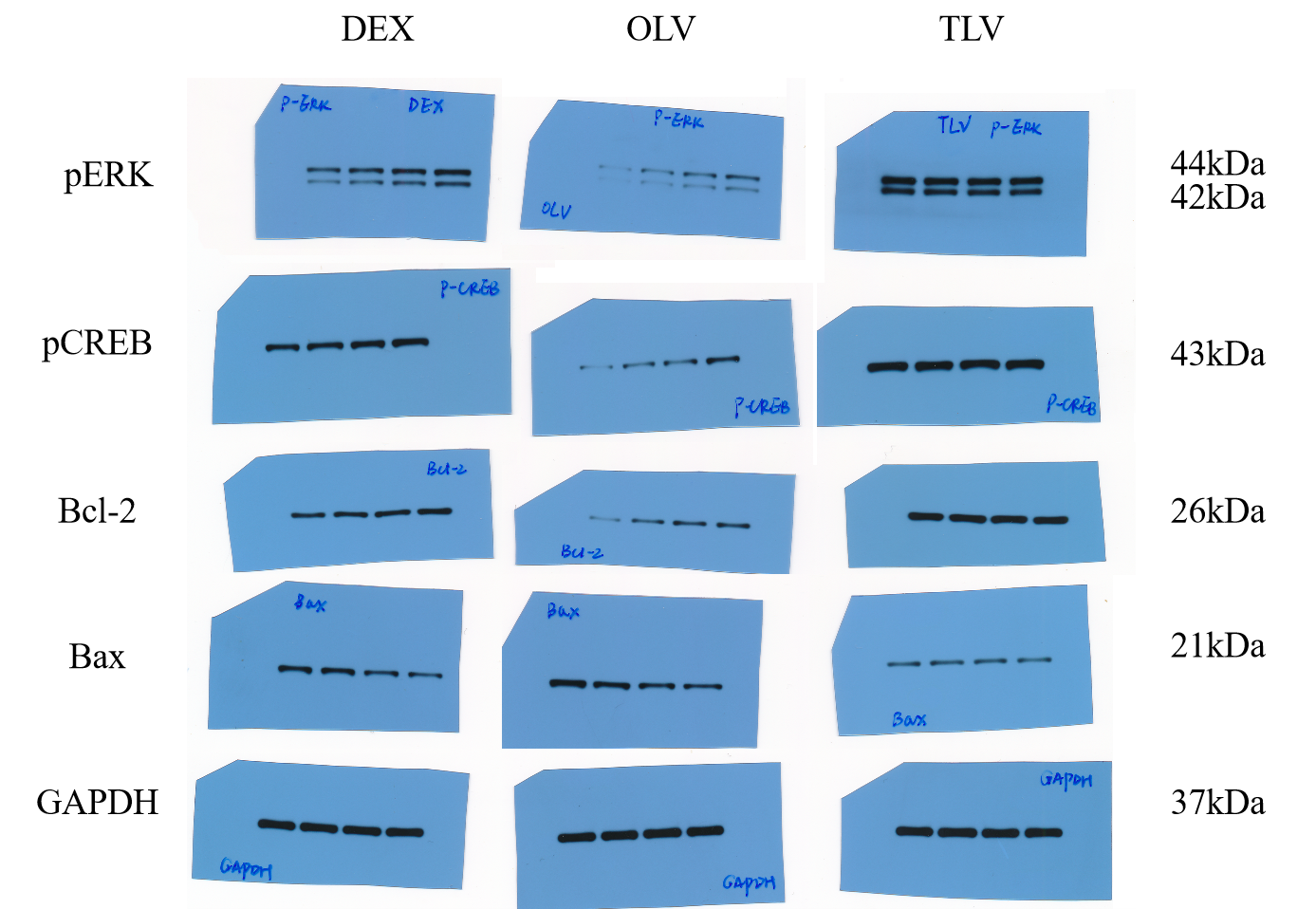

Supplement: Supplementary file 1 — Additional file 1. [file 12871_2022_1658_MOESM1_ESM.zip › original data with explanation 2.tif]

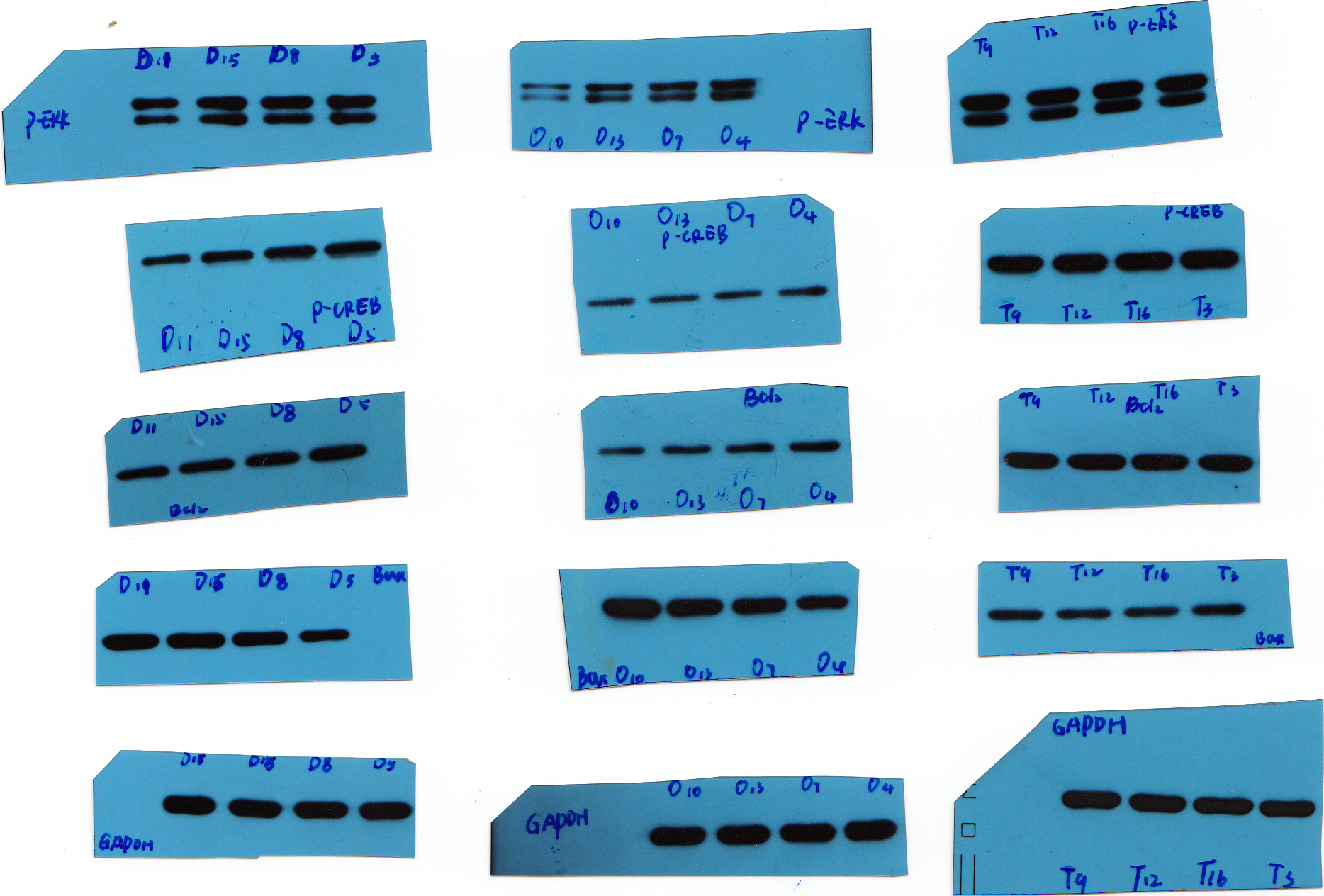

Supplement: Supplementary file 1 — Additional file 1. [file 12871_2022_1658_MOESM1_ESM.zip › WB original data.tif]
